# Characteristics of patients in platform C19, a COVID-19 research database combining primary care electronic health record and patient reported information

**DOI:** 10.1371/journal.pone.0258689

**Published:** 2021-10-19

**Authors:** Brooklyn Stanley, Andrew Davis, Rupert Jones, Steven Julious, Dermot Ryan, David Jackson, David Halpin, Hilary Pinnock, Jennifer Quint, Kamlesh Khunti, Liam Heaney, Philip Oliver, Salman Siddiqui, Anu Kemppinen, Francis Appiagyei, Emma-Jane Roberts, Antony Hardjojo, Victoria Carter, Marije van Melle, David Price, on behalf of the Platform C19 committee

**Affiliations:** 1 Optimum Patient Care, Cambridge, United Kingdom; 2 Observational and Pragmatic Research Institute, Singapore, Singapore; 3 Faculty of Health, University of Plymouth, Plymouth, United Kingdom; 4 University of Sheffield, Sheffield, South Yorkshire, United Kingdom; 5 Usher Institute, University of Edinburgh, Edinburgh, United Kingdom; 6 UK Severe Asthma Network and National Registry, Guy’s and St Thomas’ NHS Trust and Division of Asthma, Allergy & Lung Biology, King’s College London, London, United Kingdom; 7 University of Exeter Medical School, College of Medicine and Health, University of Exeter, Exeter, United Kingdom; 8 Asthma UK Centre for Applied Research, Usher Institute, The University of Edinburgh, Edinburgh, United Kingdom; 9 National Heart & Lung Institute, Imperial College London, London, United Kingdom; 10 Diabetes Research Centre, Leicester General Hospital, University of Leicester, Leicester, United Kingdom; 11 UK Severe Asthma Network and National Registry & Centre for Experimental Medicine, Queen’s University Belfast, Belfast, Northern Ireland; 12 Institute for Lung Health, Leicester National Institute for Health Research Biomedical Research Centre, University of Leicester, Leicester, United Kingdom; 13 Centre of Academic Primary Care, Division of Applied Health Sciences, University of Aberdeen, Aberdeen, United Kingdom; Monash University Malaysia, MALAYSIA

## Abstract

**Background:**

Data to better understand and manage the COVID-19 pandemic is urgently needed. However, there are gaps in information stored within even the best routinely-collected electronic health records (EHR) including test results, remote consultations for suspected COVID-19, shielding, physical activity, mental health, and undiagnosed or untested COVID-19 patients. Observational and Pragmatic Research Institute (OPRI) Singapore and Optimum Patient Care (OPC) UK established Platform C19, a research database combining EHR data and bespoke patient questionnaire. We describe the demographics, clinical characteristics, patient behavior, and impact of the COVID-19 pandemic using data within Platform C19.

**Methods:**

EHR data from Platform C19 were extracted from 14 practices across UK participating in the OPC COVID-19 Quality Improvement program on a continuous, monthly basis. Starting 7^th^ August 2020, consenting patients aged 18–85 years were invited in waves to fill an online questionnaire. Descriptive statistics were summarized using all data available up to 22^nd^ January 2021.

**Findings:**

From 129,978 invitees, 31,033 responded. Respondents were predominantly female (59.6%), white (93.5%), and current or ex-smokers (52.6%). Testing for COVID-19 was received by 23.8% of respondents, of which 7.9% received positive results. COVID-19 symptoms lasted ≥4 weeks in 19.5% of COVID-19 positive respondents. Up to 39% respondents reported a negative impact on questions regarding their mental health. Most (67%-76%) respondents with asthma, Chronic Obstructive Pulmonary Disease (COPD), diabetes, heart, or kidney disease reported no change in the condition of their diseases.

**Interpretation:**

Platform C19 will enable research on key questions relating to COVID-19 pandemic not possible using EHR data alone.

## Introduction

The COVID-19 pandemic added further burden on already stretched healthcare systems, including the routine management of patients with chronic diseases [1–4]. Primary care practitioners are especially affected as the pandemic limits their access to information regarding the health status of their patients (1).

Due to the pandemic emergency arrangements, important information gaps and inconsistencies emerged within electronic health records (EHR). Such gaps include the initial lack of codes for tests, diagnosis, and symptoms test results, remote consultations with NHS111 telephone service (the UK non-emergency medical care hotline), hospital consultations, as well as self-treatment or home testing. To understand the COVID-19-vulnerable population and clinical outcomes, key information is required on co-morbidities, medication and patient behavior such as shielding, isolation, and health-seeking behavior, information on mental health, and status of pre-existing chronic diseases.

In response to the pandemic, Observational and Pragmatic Research Institute (OPRI) Singapore and OPC (Optimum Patient Care) UK established the COVID-19 Quality Improvement (QI) program (https://optimumpatientcare.org/covid-qi/). QI programs refer to systematic sets of actions conducted to improve the quality of patient care [5, 6]. The COVID-19 QI program aims to assist primary care in assessing the incidence and impact of COVID-19 and to identify patients at-risk for COVID-19 or with suggestive symptoms who are in need of help [7]. The COVID-19 QI program also aims to increase our understanding of the pandemic such as the risk predictors of long COVID [8], the impact of the pandemic on other chronic diseases, and other potential impacts of the pandemic not yet known due to reduced healthcare consultation during the pandemic. These will be achieved by the generation of patient and practice level quality improvement reports containing key patient information combining de-identified electronic health data and patient questionnaire responses to participating practices.

Using data collected as part of this QI program, OPC established Platform C19 (https://opcrd.co.uk/platform-c19-new-2/) on September 23, 2020. Platform C19 is a novel primary care, patient-centered research platform supported by a multi-organizational research collaboration. The platform incorporates both EHR data and patient self-reported questionnaire results thereby providing a unique and comprehensive data source to facilitate multifaceted research into the COVID-19 pandemic [9].

The current article describes the development of the platform, the data collected within the platform C19, and summarizes the demographics, clinical characteristics, and behavioral patterns of the patients from the first wave of the pandemic. The impact of the pandemic on self-reported physical and social behavior, mental health, and status of chronic diseases is also described.

## Methods

### Design

This paper describes an observational, descriptive analysis of data stored in Platform C19 collected from 7^th^ August 2020 up till 22^nd^ January 2021. The analysis includes both EHR data and patient-reported information collected via questionnaire contained within Platform C19.

### Development of the platform and data collection

The OPC possesses an established track record and technical expertise in maintaining large scale research databases. We maintain the Optimum Patient Care Research Database (OPCRD), an anonymized, quality-controlled, longitudinal primary care database integrating records for more than 12 million patients from over 800 GP practices across the UK supplemented with linked patient reported outcome data from over 70 thousand patients (https://opcrd.co.uk/our-database/). Bringing our expertise, we established Platform C19 deriving EHR data from the OPCRD. OPC quality improvement programs are provided in compliance with the General Data Protection Regulation (GDPR)/Data Protection Act 2018 (Data Protection Register Ref: ZA197058) and the NHS Data Security and Protection Toolkit (Ref: 8HR85). The OPCRD has received NHS research ethics committee (REC) approval to provide anonymized data for scientific and medical research since 2010, with its most recent approval in 2015 (NHS HRA REC ref: 15/EM/0150). OPCRD is governed by the Anonymized Data Ethics and Protocols Transparency (ADEPT) committee (ADEPT1720), and the protocol of Platform C19 was approved by the ADEPT committee on 18th November 2020. The OPCRD routinely receives de-identified EHR data from GPs participating in OPC QI programs.

Access to the deidentified patient data used in this study may be requested via the OPCRD website (https://opcrd.co.uk/platform-c19-new-2/) or via the enquiries email info@opcrd.co.uk.

All patients aged 18–85 years old at the start of the pandemic (1^st^ March 2020) registered within practices participating in the COVID-19 QI program were invited in waves to complete an online COVID-19 questionnaire via text-message from their practice starting from 7^th^ August 2020. The questionnaire was designed by an expert consensus from the Platform C19 steering committee. The questionnaire covers the patient demographics, COVID-19 symptoms, diagnosis, and testing, pandemic related behavior, mental health, chronic health conditions, and detailed questions relating to asthma and COPD. The current version of the questionnaire is provided in the supplementary material. Every patient has a unique link that is attached to the electronic medical record and patient ID. This unique patient link can only be unlocked within the originating practice site. The questionnaire is sent to the patients via the medical record system of the participating practices. Responses are automatically recorded and linked to the patient ID.

Data is securely stored in an enterprise database running SQL-Server (Windows Server 2019 Standard (10.0) Version 15.0.2080.9). The list of variables collected within the platform and the sources are available in the online supplementary material (S1 Table).

To prepare data for analysis, questionnaire responses and relevant electronic medical records were collated, cleaned, and then summarized. If a patient had responded to more than one round of questionnaires, only the most recent response was used to include the most updated patient status into the analysis. This analysis includes patients who had responded to the COVID-19 questionnaire. Patients who opt-out from data-sharing for research [10] are excluded.

### Study variables

De-identified electronic health record (EHR) data and patient-reported COVID-19 questionnaire results were used in this study. Variables derived from the EHR include sex, age, frailty, and smoking status. Variables supplemented from patient-reported data include ethnicity, Body Mass Index (BMI), COVID-19 status, patient behavior, mental health status, and status of underlying chronic diseases. Asthma and COPD exacerbation were defined as requiring a course of at least 3 days of oral corticosteroid due to worsening of the condition. Respondents were considered to have had long COVID if they report symptoms lasting at least 4 weeks [11–14].

### Statistical analysis

Descriptive statistics were computed for all demographic and clinical variables using all available patient data. Categorical variables were presented as numbers and percentages. Percentages are presented according to the number of patients who answered the questions relevant to each specific variable.

## Results

### Response summary

**Fig 1:**
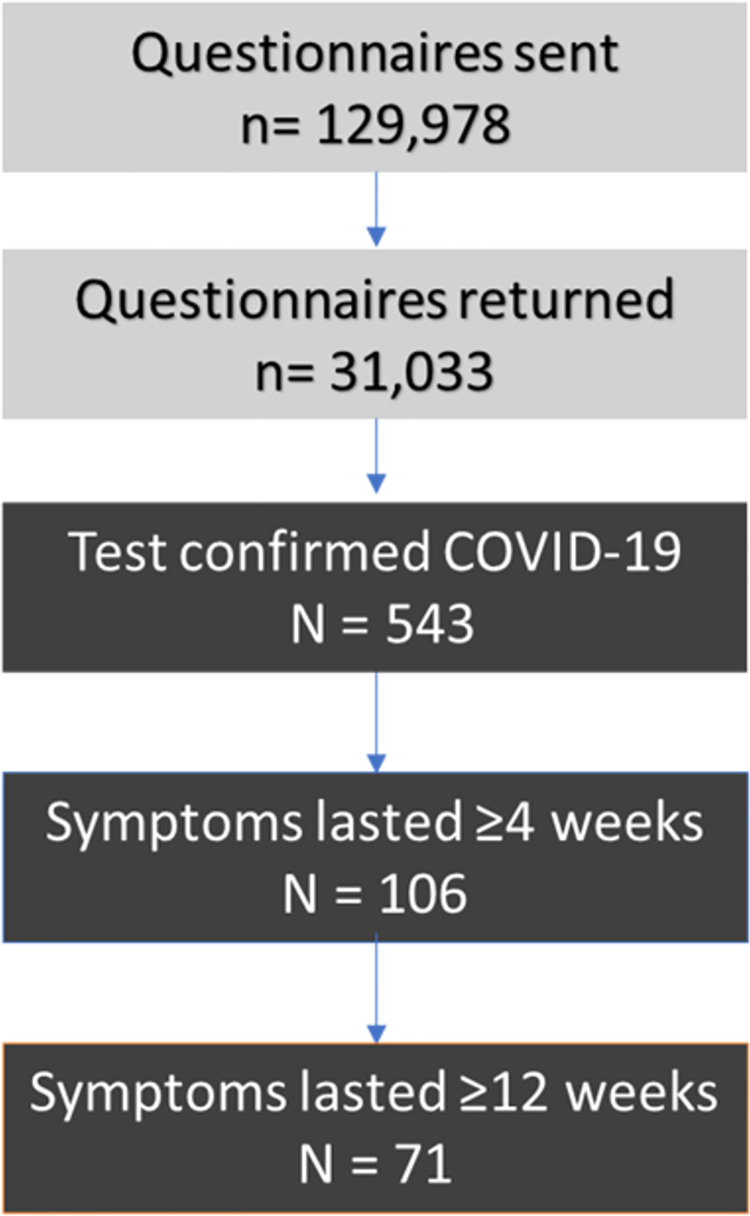
Patient flow.

A total of 129,978 questionnaire invitations were sent to all eligible patients from 14 participating general practices. Of these, 31,033 (23.9%) responded to the questionnaire (Fig 1). The average time between responses and the start of the pandemic (1^st^ March 2020) was 31.8 weeks. The average Index of Multiple Deprivation of participating practices was close to the national average with slight skew to the deprived (13231.38) and good coverage of all regions across England.

### Patient demographics and characteristics

The demographics and characteristics of the respondents are summarized in Table 1. The majority of the responding patients were female (18,504/31,033; 59.6%). Nearly all respondents were white (British, Irish, other [23,929/25,591; 93.5%]). Almost half (13,681/31,033; 44.1%) were above 60 years while 18.0% were between 18–40 years. Smoking history was common with 3,535/31,033 (11.4%) respondents reporting currently smoking and 12,792/31,033 (41.2%) previously smoking. A large proportion was also either overweight (BMI between 25.0 and 29.9 [9,966/31,033; 32.1%]) or obese (BMI >30·0 [10,945/31,033; 35.2%]). Frailty was present in 1,065/31,033 (3.4%) respondents.

**Table 1:** Demographic and clinical characteristics of survey respondents (n = 31,033).

Baseline characteristics		N (%)^a^
Sex	• **Male**	12,528 (40·4%)
	• **Female**	18,504 (59·6%)
Age group	• **18 - <40**	5,572 (18·0%)
	• **40 - <50**	4,608 (14·8%)
	• **50 - <60**	7,172 (23·1%)
	• **60 - <70**	7,408 (23·9%)
	• **70 - <80**	5,348 (17·2%)
	• **80+**	925 (3·0%)
Body mass index	• **<18·5**	397 (1·3%)
	• **18·5–24·9**	7,794 (25.1%)
	• **25–29·9**	9,966 (32.1%)
	• **30–39·9**	8,949 (28.8%)
	• **≥40**	1,996 (6·4%)
Race	• **White (British, Irish, other)**	23,929 (93·5%)
	• **Asian / Asian British**	805 (2·6%)
	• **Black / Black British**	187 (0·6%)
	• **Mixed race—White and Black/Black British**	86 (0·3%)
	• **Mixed race—Chinese / Chinese British**	96 (0·3%)
	• **Mixed race—Hispanic or Latino or Spanish origin**	74 (0·2%)
	• **Mixed race—Middle Eastern / Middle Eastern British**	61 (0·2%)
	• **Mixed race–other**	147 (0·5%)
	• **Others**	270 (0·9%)
Smoking status	• **Current smokers**	3,535 (11·4%)
	• **Ex-smokers**	12,792 (41·2·%)
	• **Non-smokers**	14,094 (45·4%)
Shielding during the pandemic	• **Shielded**	1,984 (6·8%)
	• **Not shielded**	27,203 (93·2%)
Frailty	• **No reported frailty**	29,968 (96·6%)
	• **Reported frailty**	1,065 (3·4%)
Chronic co-morbid conditions	• **Question response rate**	27,149 (87·5%)
	• **Any condition**	7,638 (28·1%)
	• **Asthma**	3,755 (13·8%)
	• **COPD, bronchitis, emphysema**	1,053 (3·9%)
	• **Diabetes**	2,358 (8·7%)
	• **Heart disease or heart failure**	1,585 (5·8%)
	• **Kidney disease**	463 (1·7%)

^a^Percentages are based on the total number of known responses.

### COVID-19 status

Testing for COVID-19 was conducted on 7,051/29,599 (23.8%) respondents, of which 543/6,904 (7.9%) reported positive test results (Fig 1). Of these, 65/543 (12.0%) were received by patients who did not have symptoms since January 2020. Long COVID, symptoms lasting 4 or more weeks, was reported by 106/543 (19.5%) positive test respondents. Further, 71/543 (13.1%) reported symptoms lasting beyond 12 weeks. More than half (362/543; 66.7%), did not seek medical attention from their GP or hospital for COVID-19 infection or symptoms. Only 120/543 (22.1%) contacted NHS 111. Around one-fifth (108/543; 19.9%) contacted their GP, of which 19/108 (17.6%) were face-to-face consultations.

### Behavior and mental health

**Fig 2:**
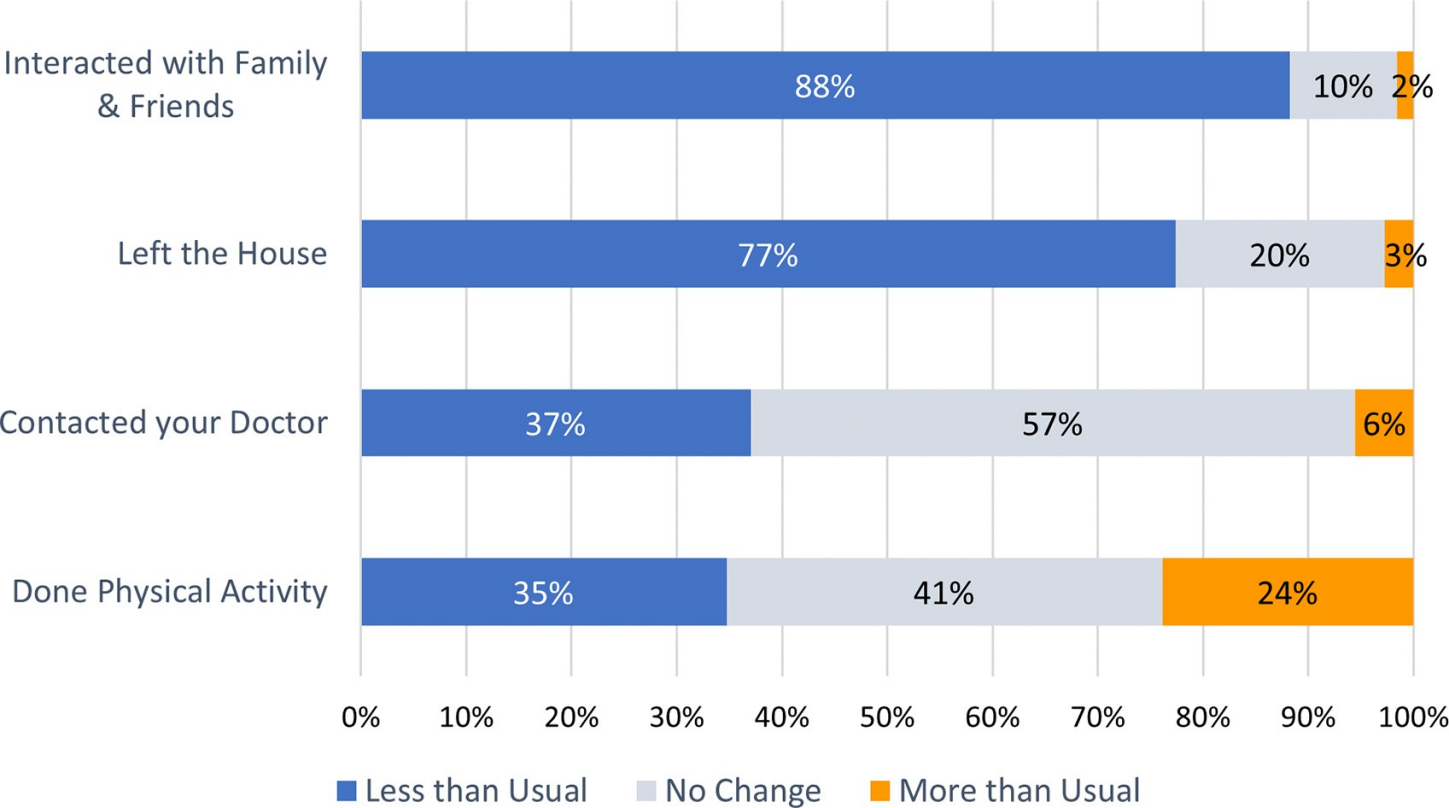
Change in respondents’ activity since March 2020.

Advice letters to shield during the pandemic was received by 2,234/29,186 (7.7%) respondents, of which 1,984/2,234 (88.8%) reported to have been shielding. A total of 1,935/27,396 (7.1%) respondents were asked to remain home for at least 14 days after travel or exposure to COVID-19. Since March 2020, most respondents reported a decrease in the frequency they interacted with family and friends (23,828 /26,992; 88.3%) or left their houses (20,503/26,723; 77.4%). No change, decrease, and increase in the frequency they contacted their doctors were reported by 15,368/26,747 (57.5%), 9,901/26,747 (37.0%) and 1,478/26,747 (5.5%) respondents respectively. While 9,094/26,181 (34.7%) reported a decrease in physical activity, another 6,250/26,181 (23.9%) reported an increase in physical activity during the pandemic (Fig 2).

**Fig 3:**
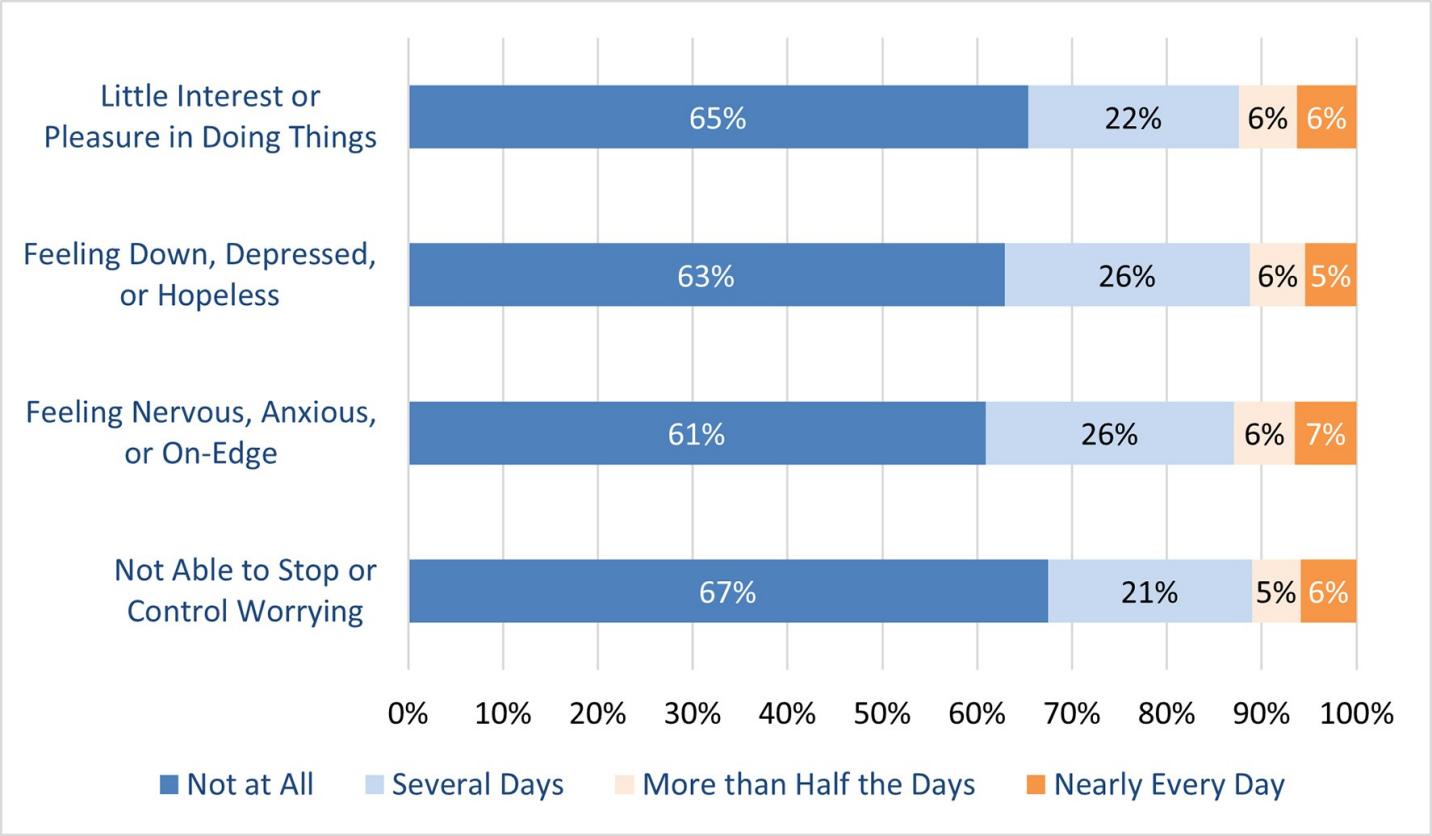
Responses to questions on mental health status in the past 2 weeks.

Up to 39% of respondents reported negative impact on question relating to their mental health status in the past 2 weeks (Fig 3). Feeling of nervousness, anxiousness, or on-edge was the most commonly reported (10,789/27,581; 39.1%) mental health problem.

### Chronic diseases status

**Fig 4:**
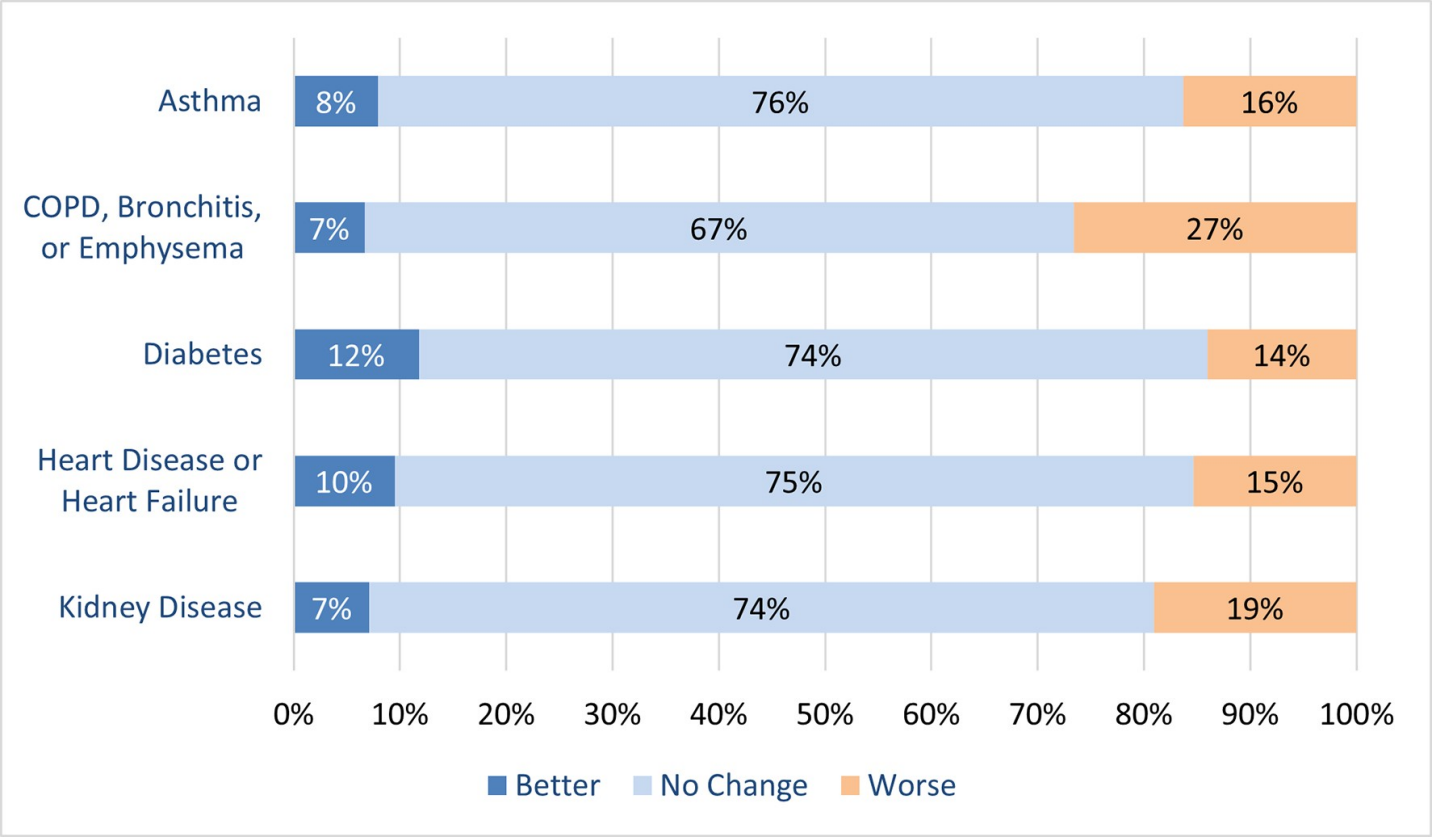
Patient-reported change in chronic conditions status since March 2020.

Chronic conditions of asthma, COPD, diabetes, heart failure, and kidney disease were reported by 3,755/27,149 (13.8%), 1,053/27,149 (3.9%), 2,358/27,149 (8.7%), 1,585/27,149 (5.8%), and 463/27,149 (1.7%) patients respectively (Table 1). Most (67–76%) respondents reported no change in the conditions of their diseases since March 2020 (Fig 4).

**Fig 5:**
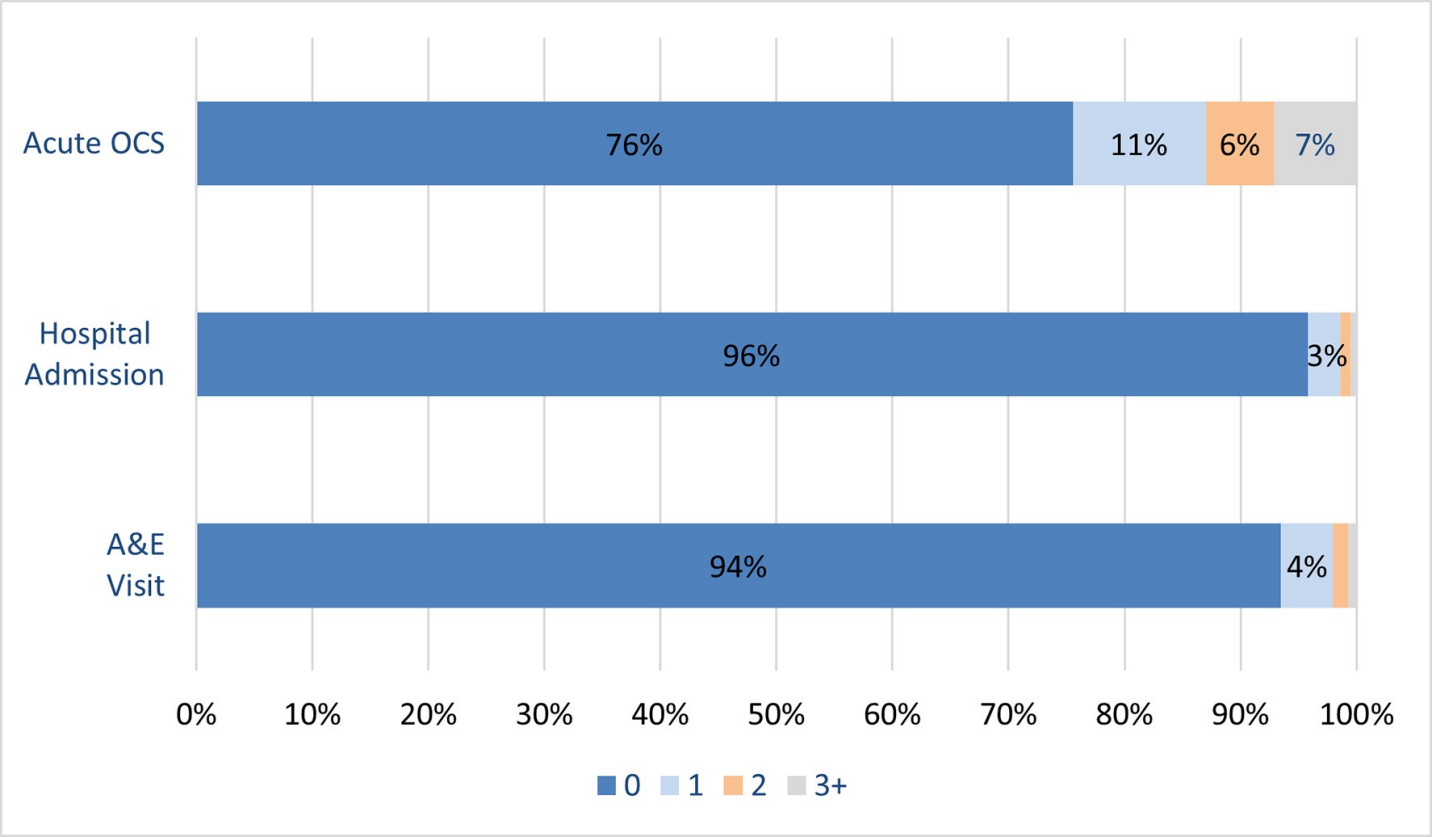
Prevalence of patient-reported markers of exacerbation of asthma and/or COPD in the past 12 months.

Most patients with asthma and/or COPD reported not having experienced any exacerbation in the past 12 months (3,162/4,185; 75.6%). Only a small proportion reported having been hospitalized (175/4,156; 4.2%) or treated in an emergency department (272/4,186; 6.5%) due to the worsening of their conditions (Fig 5).

## Discussion

### Summary of findings

In the current article, we describe the patient characteristics available within Platform C19. Platform C19 is a large population-based platform linking patient reported data linked to electronic medical records. The platform combines OPCRD’s unique access to anonymized EHR data with patient-reported information, providing additional insight on data that are not usually collected during routine clinical visits. These include data on patient behavior, potentially missed COVID-19 patients, changes in physical activity, and mental health. The platform thus provides a unique data source to answer important research questions on the COVID-19 pandemic which cannot be answered using only routine primary care EHR data.

Only a quarter of the respondents reported having been tested for COVID-19. The proportion of respondents who reported positive test results (8%) was observed to be slightly higher than the UK national data as of 22^nd^ January 2021 (56%, https://coronavirus.data.gov.uk/). Additionally, more than half reported not having sought medical attention for their COVID-19 infection or symptoms, This might have stemmed from patient reluctance to seek medical attention when they have only mild complaints. These imply that some COVID-19 cases will not be identified in EHRs.

Respondents mostly reported decreases in the frequency of interaction with family or friends and the frequency of leaving their houses since the pandemic. However, 42% and 24% of respondents reported no change or an increase in physical activities respectively despite the pandemic. This is consistent with a previous study by Spence et al that reported the majority (57%) of 1,521 adults in the UK responded to have either maintained or increased their physical activities level during the lockdown [15]. Despite this, the authors also report that the proportion of respondents who did not meet guideline recommended levels of physical activity increased during the pandemic.

Studies have consistently reported the impact of the pandemic on population mental health [16–18]. This is similarly observed among our respondents as around one-third reported some degree of negative impact in the questions regarding mental health. This observation implies a major impact of the pandemic which reaches beyond physical disturbances and highlights a need for public health policies to assist the population especially the vulnerable [19].

The pandemic disrupted the delivery of routine primary care including patients with chronic diseases [3]. However, the majority of the respondents reported no change to the status of their chronic disorders during the pandemic. Most respondents with asthma and/or COPD also reported that their symptoms had been well-controlled in the past year. These findings suggest that the majority of patients with chronic diseases may not have required face-to-face consultation with their general practitioners during the first wave of the pandemic and suggests that use of remote methods such as text message generated questionnaires or telephone reviews could support monitoring of chronic diseases including asthma and COPD.

### Further studies using data in the C19 Platform

Platform C19 is an open platform designed to facilitate collaboration and sharing of research data and resources. OPC invites collaborators to conduct crucial research on COVID-19 using the breadth of data stored in the platform.

Potential research includes analysis of the gaps in EHR recorded COVID-19 compared to the self-suspected COVID-19. Analyses of patients with self-suspected COVID-19 infection are hoped to shed information on this patient group.

Patients with chronic diseases may choose to shield during this period, which may then negatively impact their activity and quality of life [20]. Previous studies have also reported reduction in asthma and COPD related health visits since the start of the pandemic in the UK [21–23]. Whether this is due to real reductions in asthma and COPD exacerbations or due to changes in health-seeking behavior warrants further investigations. Future studies using the platform will therefore analyze the impact of shielding and other patient behavioral patterns during the pandemic towards the outcome of pre-existing chronic diseases such as asthma and COPD. Conversely, further studies will also investigate the influence of pre-existing chronic diseases on patient behavior and COVID-19 infection risk and outcome. The impact of the lockdown on the well-being of at-risk patients such as the frail, elderly, and those with multiple co-morbidities also needs to be elucidated.

Currently, there are gaps in our knowledge regarding symptoms of COVID-19 which persists after the infection, a phenomenon termed” long COVID” [24–26]. A deeper study into the subgroup of patients with long COVID identified within Platform C19 is therefore warranted to identify the potential risk factors for long COVID. This will include investigation into whether patients who self-suspected and self-managed their COVID-19 symptoms subsequently develop long COVID.

Platform C19 aims to continue data collection in the next 12 months and we expect a doubling in the number of data collected by April 2021. As the pandemic evolves, continued data collection will allow long-term follow up of patients with COVID-19. This will enable research on the impacts of new variants of the virus and the effectiveness of vaccination and other interventions including lockdowns and self-isolation.

Future rounds of questionnaire may aim to collect additional information on lockdown deconditioning (weakening of muscles due to decrease in physical activities during the lockdown), healthcare avoidance, and the impact of vaccination of their physical and mental health while subsequently increasing the representation of the Black, Asian, and minority ethnic patients within the platform. Such information will be valuable to provide recommendations for public health practitioners during a global pandemic. Platform C19 will also aim for the opportunity to link its data with secondary care data.

## Conclusions

Platform C19 is a unique research database which supplements routine EHR data with patient-reported information and outcome such as isolation behavior, mental health, and health-seeking behavior. This dataset enables researchers to answer key research questions on the pandemic which cannot be answered from routinely collected EHR data alone. We currently observe that majority of patients with chronic disease had their symptoms controlled. Respondents also reported some degree of impact on their activity and mental health due to the pandemic. Future studies conducted using Platform C19 will seek to better our understanding of the relationship between co-morbid chronic diseases, patient behavior, and COVID-19 outcome as the pandemic evolves.

## Supporting information

S1 Table Summary of key variables available within platform c19. (PDF)

S1 File COVID-19 patient questionnaire. (PDF)
