# Supplementary material for: Characteristics of patients in platform C19, a COVID-19 research database combining primary care electronic health record and patient reported information
Source: PLoS One. 2021 Oct 19;16(10):e0258689. doi: 10.1371/journal.pone.0258689 (PMC8525750; doi:10.1371/journal.pone.0258689)
Supplement: S1 Table — (PDF) [file pone.0258689.s001.pdf]

Supplementary Material

Supplementary Table 1. Summary of key variables available within platform c19.

Variable	EHR	Questionnaire
Patient consent for research		✔
Height, Weight, BMI	✔	✔
Ethnicity/Race	✔	✔
Employment		✔
Comorbid asthma	✔	✔
Comorbid COPD	✔	✔
Comorbid heart disease	✔	✔
Comorbid chronic kidney disease	✔	✔
Inhaler (adherence, technique)		✔
Asthma/COPD action plans	Partial	✔
Asthma control	Partial	✔
Asthma/COPD exacerbations	Partial	✔
COPD assessment test (CAT)	✔	✔
Biologics use for asthma/COPD		✔
COVID-19 symptoms	✔	✔
COVID-19 diagnosis	✔	✔
COVID-19 test: type, date, results	Partial	✔
COVID-19 treatment received		✔
COVID-19 treatment location		✔
COVID-19 duration		✔
Household number		✔
Exposure to COVID-19	✔	✔
Self-isolation/ Quarantine, period		✔
Shielding status, period		✔
Activity limitation, PRISMA-7 questionnaire		✔
Fitness/ frailty	✔	✔
COVID-19 related behavioral changes		✔
Depression & anxiety: PHQ & GAD	Partial	✔
Vaccination status		✔
