# Supplementary material for: Characteristics of patients in platform C19, a COVID-19 research database combining primary care electronic health record and patient reported information
Source: PLoS One. 2021 Oct 19;16(10):e0258689. doi: 10.1371/journal.pone.0258689 (PMC8525750; doi:10.1371/journal.pone.0258689)
Supplement: S1 File — (PDF) [file pone.0258689.s002.pdf]

COVID-19 PATIENT QUESTIONNAIRE

Please note that this is an online questionnaire with conditional functionality i.e. only relevant questions appear based on responses.

Introduction Page

Your questionnaire will help your practice know the impact Covid-19 is having on your health and daily life. It will help your doctor to better manage your healthcare needs. Please take a few minutes to complete the whole questionnaire.

Completion time

- The questionnaire takes about 5 mins to complete if you have not had Covid-19 or any symptoms.
- The questionnaire takes about 20 mins to complete if you have had Covid-19 or been treated for Covid-19.

1 Have you had any of these symptoms of COVID-19 since January 2020? *(Tick all that apply)*

[ ] No, I have not had any symptoms
[ ] Fever or high temperature
[ ] Persistent dry cough
[ ] Loss or change of smell or taste
[ ] Shortness of breath
[ ] Loss of appetite
[ ] Chest pain
[ ] Fatigue or tiredness
[ ] Aches and pains
[ ] Headache
[ ] Abdominal pain
[ ] Diarrhoea
[ ] Confusion or disorientation

If any symptom is selected → 1.1, 1.2

1.1 When did your symptoms start? [date]
1.2 When did your symptoms stop? [date]

2 Do you believe that you had COVID-19 infection?

[ ] Yes → 3, 8
[ ] No → 5

3 Have you been diagnosed with COVID-19 or told by a healthcare professional that you may have COVID-19?

[ ] Yes → 4
[ ] No → 5

4 How do you feel physically right now?

[ ] I feel physically normal → 5
[ ] I'm not feeling quite right → 4.1

4.1 Do you have any of these symptoms right now? *(Tick all that apply)*

[ ] Fever or high temperature
[ ] Cough
[ ] Fatigue or tiredness
[ ] Aches and pains
[ ] Abdominal pain
[ ] Diarrhoea
[ ] Shortness of breath
[ ] Loss or change of smell or taste
[ ] Loss of appetite
[ ] Chest pain
[ ] Headache
[ ] Confusion or disorientation

If any symptom is selected → 4.2

4.2 When did your symptoms start? [date]

5 Have you been tested for COVID-19?

[ ] Yes → 6
[ ] No → 11

6 6.1 Please enter the date or the month of your last test if you know it. [date]

6.2 How was this test performed?
[ ] Swab or saliva test
[ ] Blood test

7 What were the results of your COVID-19 test?

[ ] At least one of my tests was positive
[ ] None of my tests were positive
[ ] I am still waiting for my results

8 What kind of medical attention did you seek for COVID-19 infection or symptoms? *(Tick all that apply)*

[ ] No, I did not seek any medical attention
[ ] Contacted NHS 111 by phone or online
[ ] Consulted GP/practice nurse over the phone or online
[ ] Consulted GP/practice nurse face-to-face
[ ] Visited Accident and Emergency → 8.1
[ ] Admitted to hospital → 8.1
[ ] Admitted to intensive or critical care → 8.1
[ ] Other [please specify]

8.1 What treatment did you receive in hospital? *(Tick all that apply)*
[ ] Oxygen
[ ] Pressurised air delivered through face mask.
[ ] Invasive ventilation (Breathing support through an inserted tube. People are usually asleep for this procedure)
[ ] Organ support or life support
[ ] Other [enter treatment]

9 How many people are in your household including yourself?

[number]
[ ] I don't know
[ ] I live in a care home

10 Have you been exposed to someone with confirmed or suspected COVID-19 infection (such as co-workers, family members, or others)?
[ ] Yes, confirmed COVID-19 case only → 10.1
[ ] Yes, suspected COVID-19 case only
[ ] Yes, both confirmed and suspected COVID-19 cases → 10.1
[ ] Not that I know of

10.1 Have you been contacted by NHS Test and Trace?
[ ] Yes
[ ] No

11 Were you asked to remain at home for 14 days after travel or exposure to COVID-19?

[ ] No
[ ] Yes → 11.1

11.1 How many weeks or months have you self-isolated?

[number] weeks or [number] months

12 Have you been contacted by letter or text message to say you should be shielding?

[ ] No
[ ] Yes → 12.1

12.1 How many weeks or months have you been shielding and not gone out?

[number] weeks or [number] months

13 For each of the following questions please respond Yes or No:

	Yes	No
In general, do you have health problems that require you to limit your activities?	[ ]	[ ]
Do you need someone to help you on a regular basis?	[ ]	[ ]
In general, do you have any health problems that require you to stay at home?	[ ]	[ ]
If you need help, can you count on someone close to you?	[ ]	[ ]
Do you regularly use a stick, walking frame or wheelchair to move about?	[ ]	[ ]

13.1 Which one of the following best describes your level of fitness?

[ ] Fit and well (you have no active medical problems, and you exercise occasionally or regularly)
[ ] Managing well (you have medical problems that limit how active you are, but you don't need help with daily activities)
[ ] Frail (you have medical problems that limit how active you are, and you need help with daily activities and personal care)

14 Since March 2020, how often have you done the following?

	Less than usual	No change	More than usual
Interacting with family and friends	[ ]	[ ]	[ ]
Leaving the house	[ ]	[ ]	[ ]
Contacting your doctor	[ ]	[ ]	[ ]
Physical activity e.g. walking, exercise	[ ]	[ ]	[ ]

14.1 Since March 2020, have you changed your diet or medications?

	Less than usual	No change	More than usual
Your medication routine	[ ]	[ ]	[ ]
Your food and diet	[ ]	[ ]	[ ]

15 Over the last 2 weeks, how often have you been bothered by the following problems?

	Not at all	Several days	More than half the days	Nearly every day
Little interest or pleasure in doing things	[ ]	[ ]	[ ]	[ ]
Feeling down, depressed, or hopeless	[ ]	[ ]	[ ]	[ ]
Feeling nervous, anxious or on edge	[ ]	[ ]	[ ]	[ ]
Not being able to stop or control worrying	[ ]	[ ]	[ ]	[ ]

16.1 Please tell us your height in centimetres or in feet/inches:

[ ] centimetres
[ ] feet [ ] inches

16.2 Please tell us your weight in kilograms or in stone/ponds:

[ ] kilograms
[ ] stones [ ] pounds

17 Which of the following best describes your ethnicity? *(Tick all that apply)*

[ ] Asian / Asian British
[ ] Black / Black British
[ ] Mixed race - White and Black/Black British

[ ] Mixed race - other
[ ] White - British, Irish, other
[ ] Chinese / Chinese British
[ ] Hispanic or Latino or Spanish origin
[ ] Middle Eastern / Middle Eastern British
[ ] Other [please specify]
[ ] Unknown / Prefer not to say

18 Are you employed?

[ ] Employed → 18.1
[ ] Retired → 18.2
[ ] Student or in school
[ ] Unemployed

18.1 Enter your occupation or job [free text]
18.2 What was your occupation or job? [free text]

19 Do you have any of the following health problems or conditions? *(Tick all that apply)*

[ ] Asthma → 19.1 then 20
[ ] COPD, bronchitis or emphysema → 19.1 then 20
[ ] Diabetes → 19.1 then 26
[ ] Heart disease or heart failure → 19.1 then 26
[ ] Kidney disease → 19.1 then 26

19.1 Since March 2020, how well do you feel about the condition?
[ ] Better
[ ] No change
[ ] Worse

20 Are you currently on any regular inhaled medication?

[ ] No → 21
[ ] Yes → 20.1 , 20.2

20.1 How often do you take your regular inhaled medication (not your reliever inhaler)?
[ ] I take regularly (almost every day)
[ ] I take it occasionally (some days)
[ ] I don't take it any more
[ ] Not applicable

20.2 Have you had the way you take your inhaler(s) checked in the last 12 months?
Using the right inhaler technique helps you breathe the medicine straight into your lungs, where it's needed. To check and improve your inhaler technique, watch these short videos at: https://www.asthma.org.uk/advice/inhaler-videos/
[ ] Yes
[ ] No

21 Has your nurse or doctor provided you with a written plan which tells you how to recognise when your breathing condition is worsening and what you should do?
[ ] Yes
[ ] No, I have not been given advice
[ ] No written information but I have been told what to do

22 In the last 7 days, how many days:

	0	1	2	3	4	5	6	7
Have you had breathing symptoms (e.g. cough, wheeze, shortness of breath)?	[ ]	[ ]	[ ]	[ ]	[ ]	[ ]	[ ]	[ ]
Has your breathing condition interfered with your usual activities (e.g. housework, work, school)?	[ ]	[ ]	[ ]	[ ]	[ ]	[ ]	[ ]	[ ]

Have you been affected or woken by breathing symptoms?	[ ]	[ ]	[ ]	[ ]	[ ]	[ ]	[ ]	[ ]
Have you used your reliever (blue) inhaler?	[ ]	[ ]	[ ]	[ ]	[ ]	[ ]	[ ]	[ ]

23 In the last 12 months, how many times:

Have you needed a course (three or more days) of steroid tablets e.g. prednisolone or antibiotics because your breathing condition got worse? [number]

Have you been admitted to hospital because your breathing condition got worse? [number]

Have you been treated in an emergency department (A&E) or anywhere other than your GP surgery because your breathing condition got worse? [number]

23.1 If number entered > 0 → How many times since March 2020 [number]

24 These questions measure the impact your breathing condition is having on your daily life.
Please select only one answer for each question.

	0	1	2	3	4	5	
I never cough	[ ]	[ ]	[ ]	[ ]	[ ]	[ ]	I cough all the time
I have no phlegm (mucus) on my chest at all	[ ]	[ ]	[ ]	[ ]	[ ]	[ ]	My chest is completely full of phlegm (mucus)
My chest does not feel tight at all	[ ]	[ ]	[ ]	[ ]	[ ]	[ ]	My chest feels very tight
When I walk up a hill or one flight of stairs I am not breathless	[ ]	[ ]	[ ]	[ ]	[ ]	[ ]	When I walk up a hill or one flight of stairs I am very breathless
I am not limited doing any activities at home	[ ]	[ ]	[ ]	[ ]	[ ]	[ ]	I am very limited doing activities at home
I am confident leaving my home despite my breathing condition	[ ]	[ ]	[ ]	[ ]	[ ]	[ ]	I am not at all confident leaving my home because of my breathing condition
I sleep soundly	[ ]	[ ]	[ ]	[ ]	[ ]	[ ]	I don't sleep soundly because of my breathing condition
I have lots of energy	[ ]	[ ]	[ ]	[ ]	[ ]	[ ]	I have no energy at all

25 Are you given a regular injection medication (usually at hospital) also known as 'biologics' for your breathing condition?

[ ] No → 26
[ ] Yes → 25.1

25.1 Which of these injection medications do you take? *(Tick all that apply)*

[ ] Xolair (Omalizumab)
[ ] Nucala (Mepolizumab)
[ ] Cinqaero (Reslizumab)
[ ] Fasenra (Benralizumab)
[ ] Dupixent (Dupilomab)

26 Would you like to take part in future NHS approved research?

[ ] No
[ ] Yes → Please enter your email [email address]

Your information will be held securely and will only be used to contact you about research your GP practice is taking part in. For more information on how we handle and protect personal information, visit: https://optimumpatientcare.org/privacy-notice/

Thank you very much for spending time to answer these questions.
The information will be submitted to your practice to help with your care.
